# Supplementary material for: Brassica Extracts Prevent Benzo(a)pyrene-Induced Transformation by Modulating Reactive Oxygen Species and Autophagy
Source: Int J Mol Sci. 2025 Sep 29;26(19):9519. doi: 10.3390/ijms26199519 (PMC12525510; doi:10.3390/ijms26199519)
Supplement: Supplementary file 1 [file ijms-26-09519-s001.zip › ijms-3676568-supplementary.pdf]

Supplementary Figure S1

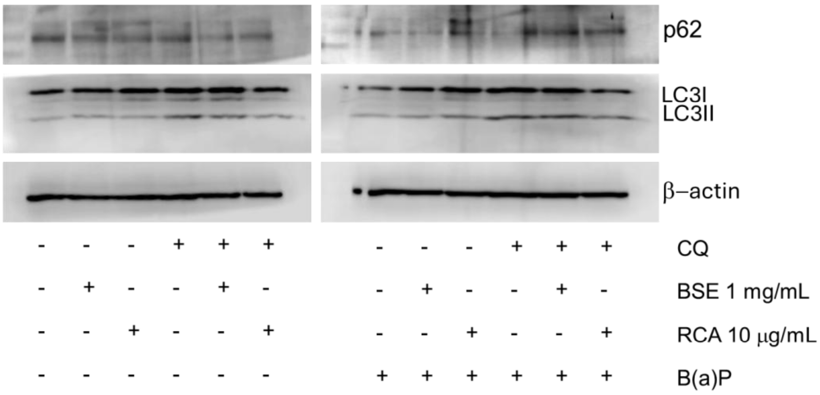

Figure S1. MCF10A cells were treated with 2 μM B(a)P or BSE/RCA extracts at the indicated concentrations for 24 h with or without chloroquine (CQ, 20 μM, last 2 h) for autophagic flux evaluation.
